# Resilience resources and coping strategies of COVID-19 female long haulers: A qualitative study

**DOI:** 10.3389/fpubh.2022.970378

**Published:** 2022-11-04

**Authors:** Atefeh Aghaei, Abhishek Aggarwal, Ran Zhang, Xiaoming Li, Shan Qiao

**Affiliations:** ^1^Department of Health Promotion, Education, and Behavior, Arnold School of Public Health, University of South Carolina, Columbia, SC, United States; ^2^South Carolina SmartState Center of Healthcare Quality, Columbia, SC, United States

**Keywords:** long COVID, female long haulers, persistent COVID-19 symptoms, resilience, coping, qualitative study

## Abstract

**Background:**

Female long haulers deal with persistent post-acute COVID-19 symptoms that have serious health implications. This study aimed to identify resilience resources at multiple socio-ecological levels for female long haulers and describe how resilience resources affect their responses to long COVID.

**Methods:**

Purposive sampling was adopted to recruit participants through social media from April to June 2021 followed by 15 semi-structured interviews. An inductive analytical approach was adopted to categorize themes by open and axial coding that were verified by peer review.

**Results:**

Female long haulers relied on resources at various socio-ecological levels to foster their resilience in response to long COVID. At the individual level, they utilized cognitive and emotional resources to increase knowledge, learn new skills, set goals, and manage emotions; behavioral resources (e.g., internal motivation and executive functioning) to perform physical, creative, and recreational activities, and adopt healthier eating habits; and spiritual resources to perform spiritual rituals and connect with God. At the social level, the support from existing relationships and/or online social support groups enhanced their social identity and provided material and informational resources. At the health systems level, the guidance from counselors and physicians and availability of clinics, medicines, and health equipment assisted them in symptom management and medication adherence.

**Conclusion:**

The resilience of female long haulers can be enhanced through (1) offering financial and health-related resources, (2) developing online social-support groups, (3) counseling and care service training for healthcare professionals, and (4) implementing more psychosocial interventions by labor organizations.

## Introduction

Today, contagious infectious diseases are one of the main threats to global public health (1). The emergence of the COVID-19 pandemic 2 years ago has affected many human lives. According to the World Health Organization (WHO) data, more than 520 million people have been infected with the novel coronavirus, with more than 6 million lives lost by May, 2022 (2). The COVID-19 pandemic also caused numerous psychological issues (3). Based on a systematic review reporting the average prevalence of COVID-19-related psychological issues on a global scale, the general public has experienced anxiety (33%), depression (28%), distress (35%), stress (40%), insomnia (32%), PTSD (3–16%), and psychological disturbance (14–72%) during the pandemic (4). Most coronavirus patients experience short-term symptoms; however, 10–15% of the patients, also known as “long-haulers,” deal with post-acute COVID-19 syndrome (PACS) (5–8). Based on the Center for Disease Control's (CDC) definition, PACS is known as post-COVID conditions (PCC), long COVID, or post-acute sequelae of SARS CoV-2 infection (PASC) and constitutes a range of new, returning, or ongoing symptoms that develop during or after COVID-19, persist for more than 12 weeks, and cannot be explained by an alternative diagnosis (9).

Long haulers suffer from serious health complications, including (1) mental health issues (10–14) such as post-traumatic stress disorder (PTSD) (15), neurological disorders and cognitive dysfunction (16, 17), attention deficit, and depression (18, 19); and (2) physical health issues such as fatigue, body aches, and difficulties in breathing (20). In addition, they also experience social and economic problems such as reduction of the social sphere, unemployment, and decreased income experienced (21, 22). Based on recent studies (9, 23–26), the risk of long COVID is approximately twice in females as compared to men. In fact, the U.S. Department of Health and Human Services (HHS) reported that females comprise up to 80% of the patient population suffering from long-lasting symptoms following infection with the virus (27, 28). The unique circumstances of females, such as intimate partner violence (IPV) (interpersonal level), caregiver roles (social level), and resource insecurity (systems level), might make them vulnerable to multi-level adversities (29–31). Intimate partner violence, i.e., psychological, physical, sexual, or economic violence, has significantly increased in quantity (35%) and severity (30%) since the start of the COVID-19 pandemic (32). Intimate partner violence had significant negative implications on females' physical and mental health due to the restrictive measures of the COVID-19 pandemic (lockdown, social isolation, social distancing). Furthermore, since women were more likely to perform “family duties” around the clock, they had lesser time for economic and work opportunities (33). The fewer economic and working opportunities not only resulted in experiencing higher IPV (33, 34), but also led in relatively poorer socioeconomic status such as lesser financial, informational, social, and emotional resources (34, 35). This multi-dimensional lack of resources can be associated with females' resource insecurity, especially the essential family expenditure for health care services.

Regarding the response of female long haulers to long COVID, resilience could play a decisive role by helping them to deal with long COVID symptoms more effectively. Resilience can be defined as the function of a person's strengths and challenges dependent on the available and accessible meaningful opportunity structure (36). The opportunity is defined as the capacity of the social and physical ecology to provide accessible resources for internal integration and external adaptation. Therefore, a person's capacity to successfully adapt to adversities that threaten health functioning is a socio-ecological process rather than an individual trait (36, 37).

Since the beginning of the COVID-19 pandemic, many empirical studies have evaluated the interplay between COVID-19 related adversities and resilience processes for different population groups. In a study on children and adolescents (38), authors found that high rates of life satisfaction (80%) among children and adolescents were due to the co-existence of psychological distress and resilience when faced with change or adversity in the pandemic. In another study on college students (39), authors showed resilience's protective role against a high loneliness rate among students during the pandemic. During the pandemic, higher levels of depression and anxiety were observed in healthcare professionals with non-resilient personality prototypes indicating the role of resilience in preserving psychological health. In this regard, appropriate information delivery, psychosocial support, treatment, and monitoring of professionals' health status were suggested to enhance resilience among healthcare professionals (40–42). Among COVID-19 patients with mild symptoms, greater levels of resilience were associated with lower depression and anxiety (43). For office workers, higher levels of resilience and positive coping strategies enhanced personal growth (44). The quarantined elders (45), similar to adults in the early months of the pandemic (46), showed higher levels of depression, anxiety, and stress when they had lower resilience levels. However, to the best of our knowledge, none of the studies have assessed coping experiences of “female-long-haulers” through the lens of a resilience framework. Thus, in this study, we aimed to identify resilience resources facilitating coping strategies used by female long haulers at multiple socio-ecological levels to mitigate the adverse impact of long COVID and describe how resilience resources affect female long haulers' response to long COVID.

## Methods

### Study design

The interview data utilized in this study were derived from an online health promotion intervention for COVID-19 female long haulers. We recruited the participants primarily by using social media, especially Facebook, through snowball and purposive sampling. In total, we recruited the participants from 16 Facebook groups, websites of two organizations related to female-long-haulers, and a Slack group. Female-long-haulers that lived in the United States and were 18 years of age or older and could speak and understand English were eligible for this study. In terms of COVID-19 experience, they should have been infected with COVID-19 with at least a persistent COVID-19 symptom for 4 weeks or longer.

After getting approval from social media groups and organizations' administrators, we posted study descriptions, the contact information of interviewers, and a recruitment flier to their websites or groups. The whole recruitment process was conducted in two and a half months, from the last week of March 2021 to mid-June 2021. We conducted semi-structured interviews with 15 out of 17 participants who completed written informed consent based on their eligibility for this study. Two independent research team members verified the eligibility of participants. The clinical diagnosis of COVID-19 and having PACS for 4 weeks or longer were checked verbally. The recruitment process continued until data saturation, when no new codes were identified from the analysis of interview data. Two researchers discussed data saturation. The sample of 15 in our study seemed sufficient for the qualitative analysis considering the recommended minimum sample size of 12 for qualitative studies (47–49). The University of South Carolina Institutional Review Board (Pro00109358) reviewed and approved the study protocol. We conducted semi-structured interviews by video conferencing software Zoom (2022). We scheduled the interviews based on the availability of interviewees and conducted them in a one-on-one online meeting including the interviewer and participant. The interviews were conducted from early April 2021 to mid-June 2021. Demographic characteristics of participants are shown in Table 1.

**Table 1:** Demographic characteristics of female-long-haulers.

**Variable**	***n* (Total = 15)**	**Percent**
**Age**		
20–35	2	13.33
36–50	6	40.00
51–65	6	40.00
>65	1	6.67
**Occupation**		
Health care provider	5	33.33
Educator	4	26.67
Business owner	4	26.67
Student	1	6.67
Retired	1	6.67
**Living situation**		
Live with others	13	86.67
Live alone	2	13.33
**Location**		
East	10	66.67
Central	3	20
West	2	13.33

### Data collection

We gathered information on the influence of persistent COVID-19 symptoms on female-long-haulers' lives and the coping strategies utilized by them. The interview guide (see Supplementary Data 1), developed for a larger study, investigates different aspects of long COVID and lived experiences of female long haulers (50). The open-ended interview questions were derived based on the literature to find similar questions that have been asked in studies of similar topics and questions theory indicates might be important. In this study, we used participants' answers to sections addressing (1) Background of COVID-19 infection and its related symptoms, (2) Psychological influences of COVID-19, (3) Coping with COVID-19 symptoms, and (4) Social support and resilience. To compensate for the time of participants, we gave a $30 Amazon e-gift card to each participant. After each interview, the interviewers wrote the field notes. The total number of interviews was mutually agreed by two researchers regarding reaching data saturation. We recorded all interviews with verbal permission from participants. The Otter.ai (2022) was employed to transcribe the recordings, and then the interviewer reviewed and verified the final texts after transcription.

### Data analysis

We utilized an inductive approach for the thematic analysis of the interviews (49, 51, 52). Our analysis comprised six stages from becoming familiar with data, constructing preliminary codes to obtaining, revising, labeling, and reporting themes (53). We used MAXQDA (2022) for analyzing the interview transcripts. For accuracy, we initially reviewed all transcripts multiple times (54, 55), and then identified the emergent themes (open coding) (56). In the next stage, we categorized identified subthemes into five main themes (axial coding). We also held team meetings to discuss the initial codes and then reorganized them to obtain the final codebook. The codebook included themes' definitions, exemplar quotes, and even quote samples not fitting into the categorization. By comparing themes, we identified differences, similarities, and interactions between themes. To check the reliability of our analysis, inter-coder agreement and peer debriefing techniques were utilized (57–59). To examine themes and outcomes, we presented final codes and findings to two team members not involved in the data analysis (57, 58). Direct quotes representing the themes were selected to explain the key findings.

## Results

### Resilience resources and coping strategies at the individual level

#### Cognitive and emotional coping strategy

Less than half of the female long haulers used their “knowledge,” “skills,” “growth mindset,” and “emotional regulation skills” to use four types of cognitive coping strategies against the PACS challenges: (1) increasing knowledge, (2) planning, (3) learning new skills, (4) realistic goal setting, and (5) emotions management.

Participants increased their knowledge on COVID-19 symptoms, treatments, and healthy behaviors through different online platforms. This information helped them reduce uncertainty about their symptoms, adopt timely and scientifically validated health-promoting actions to manage symptoms, and find resources to return to normal. One of the participants (ID#8) expressed, “*Sometimes the doctors don't know how to make you better or whatever…I tried to search to find the answer, so I've always been more research-based” (reduce uncertainty)* and the other one (ID#13) said, “*If I would have known this information about nutrition three months ago, I probably wouldn't have relapsed” (manage symptoms)*.

Participants planned new ways of living post-pandemic to enhance their well-being. They planned on “forming new relationships” and “finding new jobs.” One of female long haulers (ID#2) pointed out, “*I'm hoping to maybe open myself up to dating when the pandemic kind of dies down a little bit”* and the other one (ID#11) said “*apply for jobs…I am busy with that…participated in many interviews.”* Participants learned new skills to heal themselves and others. One of the participants (ID#9) said, “*I would like to get certified in mind-body medicine...so I think that is helping also keep my own anxiety down*” and another participant (ID#14) asserted, “*I started to learn tapping freedom technique, and that has helped and improved my health.”*

Participants engaged in setting specific and achievable goals to prioritize and tackle symptoms, and managed responsibilities based on their physical abilities. One of female long haulers (ID#5) explained, “*I try to fix one aspect of the symptoms at a time…before moving on to the next aspect*”; Similarly, one of participant (ID#12) pointed out “*I made a list of duties to do one by one.”* Additionally, they managed their physical limitations as one of the participants (ID#10) said “*I just kind of decide for each day what I'm willing to deal with and then I'm done…if I set my expectations lower... it's better.”*

All participants engaged in emotional management through engaging in sensory healing, altering their physical environments, accomplishing small tasks, becoming emotionally self-dependent, and enhancing their self-image. One of female long haulers (ID#11) mentioned, “*I started taking baths, having music in the background, lighting, candles, just being with myself. And my goal is to take care of me”* and other participant (ID#12) emphasized “*do little things to feel accomplished in self-time.”* Also, one of participant (ID#6) expressed, “*I would just need to be more emotionally secure with myself, and like, not being so hard on myself with how I'm handling it,”* and the other one (ID#3) said “*I try to just being the best version of myself…being the best mom, being the best wife.”*

#### Behavioral coping strategy

More than half of the female-long-haulers used “internal motivation” and “executive functioning skills” to adopt five types of behavioral coping strategies against the PACS challenges: (1) physical activity, (2) creative activities, (3) recreational activities, (4) healthier eating habits, and (5) volunteering.

Participants performed moderate-level mindful physical activities to regain physical fitness, improve mental health, and productively engage themselves. They engaged in swimming, walking, and yoga. One of participant (ID#4) said, “*Swimming in the pool, like was soothing to me”* and mentioned, “*I started walking…it helps lift my mood.”* Likewise, participant (ID#8) said, “*the only thing that I know, like that's helped in the past is like working out…So, I take my dog on a walk, and I love that and that kind of helps me.”* Among these activities, Yoga was the most popular relaxation technique because 13 female-long-haulers reported an increase in well-being through practice of Yoga. One of participant (ID#3) mentioned, “*It (Yoga) is therapeutic…make you feel better about yourself”* and the other one (ID#1) expressed, “*Yoga has helped me stay fluid and guided to self-awareness and rebuilding some strength in a delicate way.”*

Participants engaged in indoor and outdoor creative activities to experience immersive engagement, a state of flow, and a sense of “accomplishment.” They engaged in visual arts such as decoration, painting, and photography, and performing arts such as singing or music. One of female long haulers (ID#7) expressed, “*when my mental status or whatever is not good, and the insomnia is really bad, my release is taking pictures.”* Participants engaged in recreational activities to uplift their moods and to temporarily divert their attention from risks by watching entertaining movies and reading books. Also, one of participant (ID#2) said, “*getting lost in a book sometimes just kind of distract me.”*

Participants altered their eating habits by shifting to a healthier diet, practicing mindful eating, and eating slowly. One of female long haulers (ID#8) asserted, “*I made sure that I mindfully ate…nourish my body and helped me get better”* and the other one (ID#9) explained*, “the main thing that I've started doing now is eating less to breathing better.”* Participants adopted a new social role of “volunteering” that gave them a sense of higher purpose, meaning, and fostered their mental well-being. One of participant (ID#2) explained the “*therapeutic influence*” of volunteering, and said, “*I think that (volunteering) is helping also keep my own anxiety down.”* Also, other participant (ID#15) said*, “I participated in donating my blood so they can monitor how long the antibodies last. So, you know, and I'm interested in doing other research and helping out in other ways that I can.”*

#### Spiritual coping strategy

Half of the female-long-haulers benefitted from spiritual resources such as “spiritual rituals,” and “spiritual beliefs” to adopt three types of coping strategies: (1) performing spiritual rituals, and (2) connection with God.

Participants attended and engaged in spiritual rituals online or offline to manage stress and enhance their well-being. One of female long haulers (ID#1) asserted, “*I go to church…I feel like emotionally I'm needing more, like, soul nourishment”* and the other one (ID#15) said, “*I'll listen to one of my favorite pastors on YouTube to have peace.”* Through the spiritual beliefs, participants experienced a close connection with God that gave them a sense of protection, meaning and purpose, and induced resilience to face unpleasant situations. One of participant (ID#2) said, “*I always say prayer is when I speak to God......God's intentions are good for me, for just my life in general,”* and the other one (ID#15) mentioned, “*I just laid in bed and prayed…no worries for anything*.”

### Resilience resources and coping strategies at the social level

#### Seeking social support coping strategy

Most female-long-haulers received support from “family and friends,” “co-workers,” “therapists and counselors,” “online support groups with other patients,” “spiritual leaders and community” in the form of (1) emotional and material support, (2) informational support, and (3) stronger social identity.

The accessibility and availability of “friends and family” and “coworkers” acted as an emotional and material support system for the participants. One of female long haulers (ID#1) said, “*My friends and family have helped emotionally, physically, with food, with love, with support, with coming to clean my house, bringing flowers*.” Participants' co-workers offered a non-judgmental and a relatively “non-attached” setting to “share concerns.” One of participant (ID#8) explained, “*I feel more comfortable talking with my co-workers and supervisors than with family and friends…because my family is overprotective*.”

The healthcare providers such as therapists and counselors offered a safe space for the participants to share their concerns and enabled the participants to “learn coping skills.” One of female long haulers (ID#9) explained, “*I have probably more coping skills than most just because I had a really good therapist*.” Likewise, participants connected with other patients through online support groups, wherein, they comfortably expressed their feelings, gained meaningful information, communicated empathetically with other patients as they felt a “the sense of belongingness,” and gained access to an “advocacy group” for their rights. One of participants (ID#10) expressed, “*companionship of other patients in a similar situation made them feel that they are not alone*,” another participants (ID#6) explained, “*online support groups provide helpful information or training like belly breathing*,” and “*sharing and connecting with other patients kept me motivated*.”

Participants expressed the importance of prayer groups (spiritual community) in providing emotional and social support. One of them (ID#4) expressed, “*praying with other people help feeling not alone and to be positive.”* Another participant (ID#6) also said, “*I usually like to go to church*…*a lot of my friends from inside the church. We always feel like a team and support each other.”*

### Resilience resources and coping strategies at the health systems level

Half of the female-long-haulers used resources at the health-systems level such as “counselors,” “physicians,” “clinics,” “medicines,” and “health equipment's” to adopt three types of health management strategies: (1) symptom management, (2) medication adherence, (3) realistic social responsibilities, and (4) stress reduction by health care providers.

Participants, especially the ones with pre-existing health conditions such as pneumonia, heart disease, and geriatric conditions, consistently and actively monitored their symptoms to take preventive actions, track health progress, and improve recovery strategies. One of the participants (ID#7) asserted that “*It helps me to know when it's time to go to the E.R.,”* and the other one (ID#4) said, “*When I get short of breath, I get really anxious…a pulse ox…it's really helped my anxiety.”*

Participants reported an increase in medication adherence because they understood the severity of COVID-19 and desired a “normal” lifestyle. One of them (ID#7) asserted “*we'v*e *tried a couple of different medications...and I haven't had a headache since... that's to me is a quick win,”* and the other one (ID#5) highlighted the significant influence of medicine on enabling her to fulfill the duties and said, “*when I take medicine, then it helps me like work throughout the day and stay like focused and be able to not feel the fatigue.”*

Participants also decreased social responsibilities by presenting their true situation to friends or family. One of them (ID#8) said, “*there's something called the Spoon Theory...just so my family can kind of see where I am for the day...then I think it would have been easier for my son to understand why mom couldn't take him out.”*

Female long haulers also highlighted the role of health care providers in helping them to cope with their new health conditions. A participant (ID#10) mentioned “*I'm talking with my counselor today, you know, because we have to see our counselor, and she had a really good idea for me because I can't think for myself, you know, and diagnose myself.”* One of participants (ID#7) explained the way her therapist encouraged her to be more resilient and said “*my counselor released me from week to week. Now we're doing every three weeks - And I shared with her, I was like, ‘Victoria, this is really scary for me...and she said,’ you know, you need to depend on yourself because you are in a place where you have grown so much.”* Some of the female long haulers mentioned the stress reduction aspect of the health care providers roles. One of participants (ID#9) asserted that “*my counselor really spoke with me about one of the things that I was doing at work is when I get upset and I felt like I just needed to step away because the environment was very fast-paced*.” Also, a female long hauler (ID#7) said, “*I have a really, really good physician, that is our family physician…I do feel so comfortable with him, and he's been with me through everything. And that conversation was extremely comforting.”*

### Challenges in coping

Although female-long-haulers utilized multi-level resilience resources to cope against PCAS, they faced some challenges through the coping process, which could be new risks for their recovering from long COVID and maintaining psychosocial well-being: (1) insufficient financial resources, (2) perceived stigma against COVID survivors, (3) fear of deteriorating physical health, (4) unpleasant healthcare experiences in dealing with long COVID, (5) misinformation of long COVID, (6) overwhelming social interaction and burnout.

Most female long haulers faced shortage of money in financing treatment bills, visiting a counselor, accessing health care services, and engaging with recreational activities. The lack of sufficient finances impacted behavioral, emotional, and health management coping strategies. A participant (ID#2) asserted that “*I had counseling in my plan..., but basically stopped! Some issue is about the billing*.” Participants' perceived stigma toward their “persistent” symptoms acted as a significant barrier to adopt the social-support coping strategy. One of them (ID#8) explained “*I don't want to show that when I'm around other people because of their reactions, so I try and act like I feel good even though I may just be like short of breath and feeling absolutely crappy*.”

For more than half of the participants, the fear of deteriorating physical health acted as a barrier to adopt behavioral coping strategies such as exercising and social-coping strategies such as gathering with other people in a common place. One of the participants (ID#6) explained that “*I walk away from things, I lost a lot of friends, because I just couldn't deal with these symptoms. I had to just step back. Now I have all of these irrational fears and irrational anxiety*.”

More than half of the participants mentioned challenges in utilizing health management strategies, including “lack of access to health care services,” “lack of knowledge among doctors about COVID,” “uncertainty about treatment,” “being tired of consuming various medicine,” and “being frustrated of visiting different physician.” The challenge of identifying credible or scientifically valid information acted as a barrier for participants in “increasing their knowledge” or adopting cognitive coping strategy. Participants sometimes preferred isolation and avoiding excessive social interaction because they felt overwhelmed listening to other's issues. One of them (ID#5) asserted that “*I can't deal with other people's problems anymore because I'm almost over-empathetic now*.”

## Discussion

This study identifies and describes the coping processes and resilience resources of female long haulers. The resilience resources that facilitated coping were categorized into individual level, social level, and health systems level. The results demonstrated an interactive relationship among the multilevel resilience resources to cope with persistent COVID-19 symptoms.

**Figure 1:**
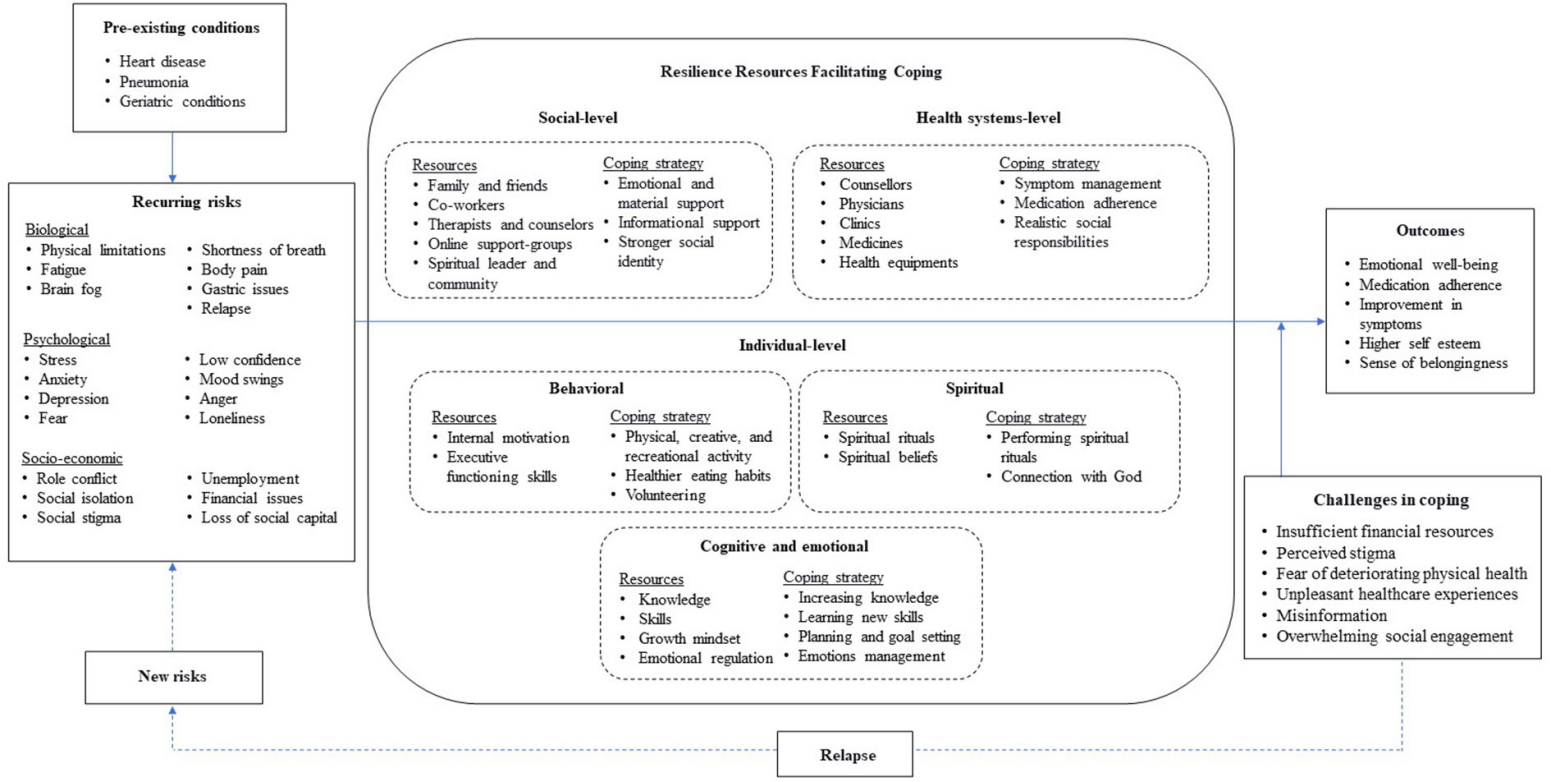
The conceptual framework of resilience resources among female-long-haulers.

The compensatory and protective models of resilience (60, 61) were adapted to connect key themes that appeared in data analysis through inductive approach. This model demonstrates resilience resources and coping strategies among female-long-haulers, and show an interactive relationship among risks, resources, and outcomes (Figure 1). In general, good outcomes refer to improvement in physical and mental health. The function of risks is negative. They may have a direct negative effect on the good outcomes and/or an indirect negative effect on the good outcomes by hampering the functioning of resources in the system. On the other hand, the function of resources is positive. A greater number of resources is positively correlated with the system's capacity to cope with adversity (62). They may have a direct positive effect on the good outcomes and/or an indirect positive effect on the good outcomes by moderating the negative impact of risks. The resilience resources of an individual, distributed across the bio-psycho-social system, facilitate coping against risks. Based on coping theory, when people encounter risks, they can use multi-level resilience resources to employ different coping strategies against risks (12). These coping strategies help to overcome, endure, or lessen the negative impact of risks (63).

In the context of long COVID, risks toward outcomes were at three levels i.e., biological, psychological, and socio-economic in addition to the pre-existing conditions (e.g., various chronic diseases). The resilience resources at multiple socioecological levels could mitigate the influence of risk factors on mental health outcomes of female-long-haulers through promoting adaptive and positive coping strategies. However, the performance of these resources could change in the process of psychosocial adaptation to chronic illness (64, 65). The common individual-level resilience resources include self-efficacy (mastery motivation) (66–68), self-regulation skills (emotional regulation) (69–71), cognitive abilities (72, 73), hope and meaning (74), religious faith (75), and personality trait (76). An individual's inner strengths and resilience always interact with external factors within a particular social-ecological context. For example, interpersonal resilience resource may include family support (77), peer group and social network (78), neighborhood (79, 80), and structural level factors include policies and institutions (81, 82), and culture (83).

Other studies also used compensatory and protective models of resilience to study resilience in the context of COVID-19. The compensatory model of resilience has been used to understand the relationship between resilience and anxiety among pregnant women during the COVID-19 pandemic (84). Consistent with our results, the authors showed that pregnant women with adaptive profiles could better adapt to changes in their social life and often have higher self-efficacy and coping abilities. They demonstrated that these individual-level factors might develop resilience in pregnant women by providing more resources to cope with stressors. In another study based on protective model of resilience, in line with our study, authors found that enacting the two coping mechanisms (Problem-focused and Emotion-focused strategies) and resilience resources is essential to achieve an adaptive effect on health practitioners' mental health (85).

As stated by participants, relapse of symptoms could occur in long Covid. Given that long Covid includes various symptoms across organ systems, such as shortness of breath, fatigue, and cognitive impairment, we observed cycles of wellness followed by relapse in long haulers (86). Davis et al. (87) reported that 86% of people with long COVID could experience relapses that often occur in an irregular pattern or in response to triggers like stress, physical or mental activity, heat, menstruation, or alcohol (87).

As stated in our results, participants reported various types of coping strategies. They tapped on their internal resources (intrinsic motivation, executive functioning skills, knowledge, skills, and positive attitude) to adopt new behaviors, emotionally regulate themselves, and seek social support. These findings are consistent with other studies which identified that meditation (88, 89), dietary changes, recreational activities, and physical activity were associated with distress reduction (90) and had antidepressant properties (91) and improved symptoms among COVID-19 patients (92). Emotional regulation reduced anxiety (93), depression (11), and emotional distress (94) during the COVID-19 pandemic. People in other disasters, like SARS, also used this coping strategy (74, 95, 96). Seeking and accessing to social support from others, especially from healthcare workers and other patients could also act as adaptive coping strategies during the COVID-19 outbreak. It is notable that participants reported taking on new social roles (e.g., volunteers to support other patients) as a unique coping strategy. Volunteer work has been shown to have the potential to facilitate the recovery of psychological well-being and allow individuals to enhance their social lives (97). Although this coping strategy is recognized as a new one among female-long-haulers, it has been employed among some populations, like nurses dealing with COVID-19 patients, whose occupation requires care for others. Helping others, in general, can be regarded as a form of self-help, leading to higher levels of confidence and self-awareness, with a decrease in depression (98).

Though common coping strategies were embedded at the individual level, the resilience resources that facilitated this coping went beyond the individual. Many participants reported the use of resilience resources at the social and systems level to cope with long COVID similar to other studies (99, 100). At the health-systems level, the affordability, availability, and accessibility to physical and mental health care providers, along with trustworthy medical equipment and resources, acted as a resilience resource for the participants. The health care providers not only contributed to symptom management and treatment, but offered emotional, cognitive, social, and behavioral support (101, 102). Participants reported an increase in medication adherence, symptom management, knowledge about long COVID, hope for better physical health, and better management of social responsibilities.

At the social-level, apart from the support of close friends, family, and co-workers, participants reported the online or social-media based support groups as an important resilience resource. Our findings showed that participants discussed their symptoms, potential treatments, and other COVID-related information with other long haulers through online support groups. The right amount of privacy and anonymity enabled participants to share freely. Participants highlighted that although they faced physical or mental limitations for socializing with different people, the online social support groups enabled them to tackle loneliness. Additionally, participants experienced emotional comfort as someone else was experiencing similar issues. Some participants also reported gaining access to material resources through the online support groups. Some studies mentioned that some patients checked their health status during their infection and shared some of their symptoms on social media in order to raise awareness or find a solution to their problem (103). Although other studies highlighted the role of online support for educating students (104, 105) or searching for information about COVID-19 (106, 107), the use of online support groups for multi-level coping with persistent COVID-19 symptoms is a new finding represented in this study.

Certain factors limited this study results. The small sample size of participants i.e., 15 participants, did not allow for investigating the role of key demographic variables, which would have allowed us to assess the potential influence of race or ethnicity on social life of female-long-haulers. However, this type of qualitative research that is typically based on small samples, seek to provide trustworthiness and sufficient context to a greater extent to allow readers to make their own judgment regarding transferability (108). Like all other qualitative research, our study may have also been influenced by the researchers' subjective bias during the process of guide development, transcription coding, and result interpretation. Furthermore, this study was part of an online health intervention among long haulers and recruited all potential participants online, particularly from Facebook groups. Hence, members of our sample were the individuals already interested in online intervention and had access to social media which limited their ability to be a representative of the general population. Also, not obtaining the proof required to verify the clinical diagnosis of COVID-19 and PACS for participants may limit our results' applicability for future clinical studies.

Despite these limitations, our findings demonstrate the importance of resilience resources for female-long-haulers' coping process. It is critical to identify and capitalize the fundamental resilience resources of female-long-haulers as they persistently impact the health outcomes over a long period of time through dynamic channels (109). This study also shows a demand for work organizations to plan for and implement more psychosocial measures to provide their staff a healthy coping with COVID-19. Healthcare professionals and social workers can capitalize on social and system level resilience resources to facilitate coping against long COVID among female-long-haulers. First, there is a need to develop and regulate online support groups to allow female-long-haulers to share health-related information and express their feelings freely. Second, at the policy-level, financial interventions for facilitating access to healthcare services and insurance is necessary as many female-long-haulers reported the lack of finances as a major barrier to cope with long COVID. Third, as the study indicated the important role of healthcare provider in offering multi-level coping strategies, the healthcare providers can offer a pool of resources to female-long-haulers such as information on online support groups, techniques for managing social responsibilities, and online channels to gather trusted information when required. Overall, the healthcare professionals need to facilitate coping in female long haulers from the intersectionality lens, adopting policies to balance job/family roles to promote gender equality in healthcare services and the job market for COVID-19 female-long-haulers.

## Conclusion

This study identified and described the resilience resources and coping strategies for female-long-haulers in the United States to mitigate the detrimental effects of long COVID on their mental health. Although some coping strategies such as physical and recreational activity, emotional management, social support, and shifting to a healthier diet were common among female-long-haulers, some others, like cognitive and spiritual coping strategies, were rarely considered. Our findings confirmed the prominent role of family, friends, and support groups as powerful resilience resources. Moreover, the current study revealed some novel resilience resources and related coping strategies among female-long-haulers, including health management, taking social roles, and online social support. Health care providers and counselors had a unique position since their roles may promote resilience among female-long-haulers by providing more positive orientation and helping to practice new stress reduction techniques.

## Data availability statement

The raw data supporting the conclusions of this article will be made available by the authors, without undue reservation.

## Ethics statement

All methods were carried out in accordance with guidelines of qualitative study. This study was reviewed and approved by the University of South Carolina Institutional Review Board (Pro00109358). The informed consent was obtained from all the participants prior to the interview.

## Author contributions

SQ contributed to the study conception and design. Material preparation and data analysis were performed by AAgh and AAgg. The first draft of the manuscript was written by AAgh, and AAgg. RZ and SQ also made major contribution in manuscript writing. SQ and XL have reviewed and revised the manuscript. All authors read and approved the final manuscript.

## Funding

Research reported in this publication was supported by the National Institutes of Health under Award #R01AI127203-5S1.

## Conflict of interest

The authors declare that the research was conducted in the absence of any commercial or financial relationships that could be construed as a potential conflict of interest.

## Publisher's note

All claims expressed in this article are solely those of the authors and do not necessarily represent those of their affiliated organizations, or those of the publisher, the editors and the reviewers. Any product that may be evaluated in this article, or claim that may be made by its manufacturer, is not guaranteed or endorsed by the publisher.

## Author disclaimer

The content is solely the responsibility of the authors and does not necessarily represent the official views of the National Institutes of Health.
