# Supplementary material for: Resilience resources and coping strategies of COVID-19 female long haulers: A qualitative study
Source: Front Public Health. 2022 Nov 4;10:970378. doi: 10.3389/fpubh.2022.970378 (PMC9672809; doi:10.3389/fpubh.2022.970378)
Supplement: Supplementary file 1 [file Data_Sheet_1.docx]

**Interview guide**

**Hi.  My name is _______, I am a graduate student/post-doc at the department of xxx of USC. As one of the interviewers for the COVID-19 study, I will be conducting this interview today. I would first like to thank you for taking the time to talk with me today—your thoughts and opinions are very important, and I want to thank you for being here.**

**This interview is focused on your experiences and feelings of long COVID symptoms, the impacts of these symptoms on your life, your coping strategies, and your needs and opinions about a potential intervention.**

**For any question I ask, there is no right or wrong answer. I appreciate anything you are willing to share and am looking forward to learning from you. Our talk is confidential. I would not be able to discuss anything we have talked about with you in public. All your responses will be destroyed upon the completion of our study.**

**This interview should take around 45 minute to complete. I will turn my phone to silent during the interview- would you like to do the same?  If you need to take a break at any time, just let me know. Are there any questions I can answer? Do you have anything you need to do before we start?**

*If yes, answer participant’s questions.*

**I will now start recording. These recordings get transcribed into text and when we do that, we remove any information, like names, that could identify someone. Documents from all our interviews then get read and re-read to identify the main points people have made. I may take some notes during the interview so I can remember what you are telling me, and these notes will be destroyed after the interview.**

**First, can you briefly describe yourself? Like age, the state you are living in, your occupation, etc.**

| **Domains/checklist** | **Example Questions** | **Decision** |
| --- | --- | --- |
| **I. Background of COVID-19 infection and its related symptoms** | 1. When were you diagnosed with COVID-19?  2. What symptoms have you experienced since the infection?  3. Do you experience any long-term (or persistent) symptoms, which last for weeks or even months after recovery?  4. Do these symptoms affect your life? (social, working, daily life, etc.)  5. What are the biggest challenges that the COVID-19 infection and long-COVID symptoms brought to you? |  |
| **II. Psychological influences of COVID-19** | 1. Did the symptoms effect your moods? Your mental health? (describe some examples)  2. Do other people know that you have these symptoms?    3. How do they respond it? (Describe specific examples and/or situations) | 2. If no, move to III. |
| **III. Coping with COVID-19 symptoms** | 1. What have you done to deal with the symptoms? (e.g., emotionally settle down, manage the symptoms, health seeking, information seeking)  2. Have you tried any approaches to reduce stress related to COVID-19? Would you like to share your experience?    3. Which approaches do you find effective to release your distress?  4. Where do you learn these approaches? | 2. If no, move to IV |
| **IV. Social support and resilience** | 1. Who do you turn to when you experience stress and distress? How did they help?  2. Do you need any additional resources or help to enhance your physical and mental health? If so, what are they?  3. Are there any things you do to help to overcome bad feelings and bounce up? |  |
| **V. Mindfulness practice** | 1. Do you know about mindfulness?  2. Have you practiced mindfulness or meditation before?  3. Tell me more about your experiences (e.g., when started it? When/where do you practice it? What do you usually practice mindfulness for? How frequently do you practice mindfulness? Do you it works?)  4. Have you ever practiced mindfulness or meditation to manage stress or symptoms since your infection? Tell me more about your experiences. | 1. If no explain mindfulness and skip to VI. |
| **VI. Attitudes towards an online mindfulness-based intervention** | *Interviewer explains proposed Mindful Walking Intervention prior to questions in this section*  1. Are you interested in joining mindfulness self-care intervention? Why or why not?  2. What is your expectation from this intervention?  3. Any suggestions or concerns about the intervention? (e.g., format, number of sessions, which social platform we should post flyers, etc.)  4. Would other persons infected with COVID-19 accept this program? Why or why not? |  |
| **VII. Others** | 1. Do you have any questions for me about the study and intervention?  2. Is there anything else would you like to share with me? |  |
